# Proof of Gene Doping in a Mouse Model with a Human Erythropoietin Gene Transferred Using an Adenoviral Vector

**DOI:** 10.3390/genes12081249

**Published:** 2021-08-16

**Authors:** Takehito Sugasawa, Takuro Nakano, Shin-ichiro Fujita, Yuki Matsumoto, Genki Ishihara, Kai Aoki, Koki Yanazawa, Seiko Ono, Shinsuke Tamai, Lev Manevich, Haruna Ueda, Noriyo Ishibashi, Kenshirou Tamai, Yasuharu Kanki, Yasuko Yoshida, Koichi Watanabe, Tohru Takemasa, Yasushi Kawakami, Kazuhiro Takekoshi

**Affiliations:** 1 Laboratory of Clinical Examination/Sports Medicine, Division of Clinical Medicine, Faculty of Medicine, University of Tsukuba, 1-1-1 Tennodai, Tsukuba 305-8577, Japan; take0716@krf.biglobe.ne.jp (T.S.); shin.ichiro.fujita.03@gmail.com (S.-i.F.); K-Aokitsuku@md.tsukuba.ac.jp (K.A.); yasukk1220@gmail.com (Y.K.); y-yoshida@tius.ac.jp (Y.Y.); y-kawa@md.tsukuba.ac.jp (Y.K.); 2 Graduate School of Comprehensive Human Sciences, University of Tsukuba, 1-1-1 Tennodai, Tsukuba 305-8577, Japan; s2021408@s.tsukuba.ac.jp (T.N.); s1921312@s.tsukuba.ac.jp (K.Y.); jimbeamilkybonbon330@gmail.com (S.O.); tama1994@outlook.jp (S.T.); lew.manewitsch@gmail.com (L.M.); 3 Research and Development Section, Anicom Specialty Medical Institute Inc., 2-6-3 Chojamachi 5F, Yokohama 231-0033, Japan; ymatsumoto.ac@gmail.com (Y.M.); genki.ishihara@ani-com.com (G.I.); 4 Japan Society for the Promotion of Science, Kojimachi Business Center Building, Kojimachi, Chiyoda-ku, Tokyo 102-0083, Japan; 5 Department of Experimental Pathology, Faculty of Medicine, University of Tsukuba, 1-1-1 Tennodai, Tsukuba 305-8577, Japan; 6 Analyst-Accenture Technology, Intelligent Platform Services, Accenture Japan Ltd., Akasaka Intercity AIR, 1-8-1 Akasaka, Minato-ku, Tokyo 107-8672, Japan; hanamoo1995@gmail.com; 7 Tsukuba i-Laboratory LLP, 2-1-17 Amakubo, Tsukuba 305-0005, Japan; ishibashi@tsukuba-i-lab.com (N.I.); tamai@tsukuba-i-lab.com (K.T.); 8 Department of Medical Technology, Faculty of Health Sciences, Tsukuba International University, 6-20-1 Manabe, Tsuchiura 300-0051, Japan; 9 Faculty of Health and Sport Sciences, University of Tsukuba, 1-1-1 Tennodai, Tsukuba 305-8574, Japan; watanabe.koichi.ga@u.tsukuba.ac.jp (K.W.); takemasa.tohru.gm@u.tsukuba.ac.jp (T.T.)

**Keywords:** gene doping, gene therapy, erythropoietin, adenoviral vector, sports, athlete, RNA sequencing

## Abstract

Despite the World Anti-Doping Agency (WADA) ban on gene doping in the context of advancements in gene therapy, the risk of *EPO* gene-based doping among athletes is still present. To address this and similar risks, gene-doping tests are being developed in doping control laboratories worldwide. In this regard, the present study was performed with two objectives: to develop a robust gene-doping mouse model with the human *EPO* gene (h*EPO*) transferred using recombinant adenovirus (rAdV) as a vector and to develop a detection method to identify gene doping by using this model. The rAdV including the h*EPO* gene was injected intravenously to transfer the gene to the liver. After injection, the mice showed significantly increased whole-blood red blood cell counts and increased expression of hematopoietic marker genes in the spleen, indicating successful development of the gene-doping model. Next, direct and potentially indirect proof of gene doping were evaluated in whole-blood DNA and RNA by using a quantitative PCR assay and RNA sequencing. Proof of doping could be detected in DNA and RNA samples from one drop of whole blood for approximately a month; furthermore, the overall RNA expression profiles showed significant changes, allowing advanced detection of h*EPO* gene doping.

## 1. Introduction

Doping is the act of using prohibited substances and/or methods in sports to enhance athletic performance and success by improving physical performance [1]. The World Anti-Doping Agency (WADA), which was established in 1999, is involved in scientific research on doping, anti-doping education, development of anti-doping strategies, and monitoring of the World Anti-Doping Code to ensure soundness and fairness in sports worldwide [2]. However, despite WADA’s substantial efforts, doping has not been eradicated from competitive sports.

The International Standard Prohibited List [3] stipulated in the World Anti-Doping Code 2021 [4], which is published with annual revisions by the WADA, describes various formulations and methods used in doping. This list includes “gene doping” as a form of abuse of gene therapy technology. Therefore, our research group has been conducting research for more than five years to establish an examination method for gene doping [5,6,7].

Gene therapy has rapidly evolved as a new treatment method for single-gene hereditary diseases and acquired diseases, and these treatments have yielded many beneficial results [8]. In fact, gene therapeutics, as represented by Collategen (AnGes, Ibaraki, Osaka, Japan) for arteriosclerosis obliterans and Buerger’s disease, Zolgensma (Novartis Gene Therapies, Chicago, IL, USA) for spinal muscular atrophy, and YESCARTA (Kite Pharma, Santa Monica, CA, USA) for relapsed/refractory large B-cell lymphomas, have been approved for the treatment of various human diseases. Recently, gene therapy technology has also been used to develop vaccines based on mRNA [9] or adenoviral vectors [10] against coronavirus disease 2019 (COVID-19) caused by severe acute respiratory syndrome coronavirus-2 (SARS-CoV-2). The substantial increase in research on gene therapy since the early 2000s led the WADA to raise concerns about the abuse of gene therapy technology for doping. However, the development of robust and universal methods to identify gene doping is still ongoing. Although WADA recently established laboratory guidelines for PCR methods in gene-doping tests, and the guidelines describe basic validation for the PCR methods in sufficient detail [11], they do not include descriptions of detection methods for individual target genes or vectors. Thus, there is an urgent need to develop detailed detection methods that can detect any type of gene doping.

The erythropoietin (*EPO*) gene has been identified as a target for abuse in gene doping because EPO- and EPO-related formulations have been frequently used in doping to enhance the endurance performance of athletes in cycling, boxing, athletics, and rowing [12,13,14,15,16], and EPO and EPO-related formulations are included on the International Standard Prohibited List [3] published by WADA. According to the Wiley database on Gene Therapy Trials Worldwide [17], recombinant adenovirus (rAdV) is the most frequently used vector in clinical trials for gene therapy. Thus, there is a strong possibility that combinations of the *EPO* gene and rAdV are being used for gene doping in human athletes, necessitating methods for detecting gene doping using this combination.

Although some previous studies have attempted to establish animal models of gene doping by using naked plasmid vectors, including the *EPO* gene [6,18,19], they did not evaluate the phenotypic effects of *EPO* gene doping, such as changes in the number of red blood cells (RBCs), gene and protein expression of EPO, and secretion levels of EPO in the blood. For these reasons, these models showed limited utility for the development of detection methods for gene doping. Moreover, none of these reports established a gene-doping model using a combination of the *EPO* gene and rAdV. On the basis of these previous studies and history, we recognized that a robust model for developing a detection method for direct or indirect proof of gene doping using rAdV and the human *EPO* (h*EPO*) gene (rAdV-h*EPO*) was needed. As direct proof, we considered that the detection of viral vector-specific DNA fragments by a TaqMan qPCR assay would be the most reasonable approach because the principle underlying this assay has already been successfully used in testing for SARS-CoV-2. In addition, as indirect proof, we considered that monitoring fluctuations of RNA expression in whole-blood samples could be applied to the parameters of the Athlete Biological Passport (ABP) because the fundamental principle underlying the ABP involves monitoring selected biological variables that indirectly reveal the effects of doping over time rather than attempting to detect the doping substance or method itself [20]. Furthermore, the WADA describes that the ABP can be used to identify athletes requiring further attention through intelligent, timely interpretation of passport data and can notably be used as a complement to analytical methods to further refine and strengthen overall anti-doping strategies [20]. In other words, the detection of indirect proof in gene-doping tests is also important to identify athletes requiring further attention and to support the findings determined using direct proof. Therefore, in this study, total RNA sequencing (RNA-seq) using whole-blood RNA from an animal model was also performed to identify RNAs as a form of novel indirect proof based on the concept of the ABP.

In summary, the primary objectives of this study were the establishment of a robust gene-doping mouse model using rAdV-h*EPO*, the identification of RNA as indirect proof based on the concept of the ABP, and the development of detection methods to obtain direct and indirect proof for gene doping.

## 2. Materials and Methods

### 2.1. Creation of the rAdV-hEPO Vector

To establish a robust gene-doping mouse model injected with rAdV-h*EPO*, a viral vector was created using the following procedures. Three plasmids, namely, (p) h*EPO*-Myc-DDK-tag (Cat# RC210775L1; ORIGENE, Rockville, MD, USA), pENTR4 (Cat# A10465; Thermo Fisher Scientific, Waltham, MA, USA), and pAd/CMV/V5-DEST (Cat# V49320; Thermo Fisher Scientific, Waltham, MA, USA; can make rAdV type 5), were used to create the rAdV-h*EPO* vector. First, PCR was performed to amplify the h*EPO* gene, including the myc and DDK (FLAG) tags and the restriction enzyme sites of *EcoR*I/*Not* I from the ph*EPO*-Myc-DDK-tag by using PCR enzymes (KOD-Plus-, Cat# KOD-201; TOYOBO, Osaka, Japan). The PCR amplicon was subjected to gel electrophoresis, after which the amplicon DNA was extracted and purified from the gel. After digestion of the purified amplicon and pENTR4, a homologous recombination reaction was induced in the mixture by using an In-Fusion HD Cloning Kit (Cat# 63964; Takara Bio, Kusatsu, Shiga, Japan). The obtained plasmid (pENTR4-h*EPO*) was mixed with pAd/CMV/V5-DEST, and an LR recombination reaction was induced using Gateway LR Clonase II Enzyme Mix (Cat# 11791020; Thermo Fisher Scientific, Waltham, MA, USA). Finally, the obtained plasmid (pAd/CMV/V5-DEST-h*EPO*) was digested using a *Pac*1 restriction enzyme (Cat# R0547; New England Biolabs, Ipswich, MA, USA) and transfected into HEK 293A cells (Cat# R70507; Thermo Fisher Scientific, Waltham, MA, USA) with a transfection reagent (PEI MAX, Cat# 24765-1; Polysciences, Warrington, PA, USA) to amplify rAdV type 5 including the h*EPO* gene cassette. The amplified rAdV-h*EPO* vectors were purified by CsCl density-gradient ultracentrifugation followed by gel filtration with a PD-10 Column (Cat# 17085101; Cytiva, Marlborough, MA, USA) according to the protocol described by Takeuchi et al. [21,22]. The viral particles (VPs) were suspended in 10% glycerol/phosphate-buffered saline (PBS), and the concentration of rAdV VPs was measured using a spectrophotometer according to the method described by Sweeney and Hennessey [23].

### 2.2. Animal Experiments

All animal experiments in this study were approved by the Animal Care Committee, University of Tsukuba (approval number: 20-361). Seven-week-old male ICR mice were purchased from CREA Japan (Meguro, Tokyo, Japan) and then subjected to a 1-week acclimation period. The mice were bred and maintained in an air-conditioned animal house under specific pathogen-free conditions and subjected to a 12/12 h light/dark cycle. The mice were fed standard mice pellets and water ad libitum. At the start of the experiments, the age of the mice was 8 weeks. The animal experiments were broadly divided into short- and long-term experiments. None of the mice died during the experimental procedures described below.

**Figure 1:**
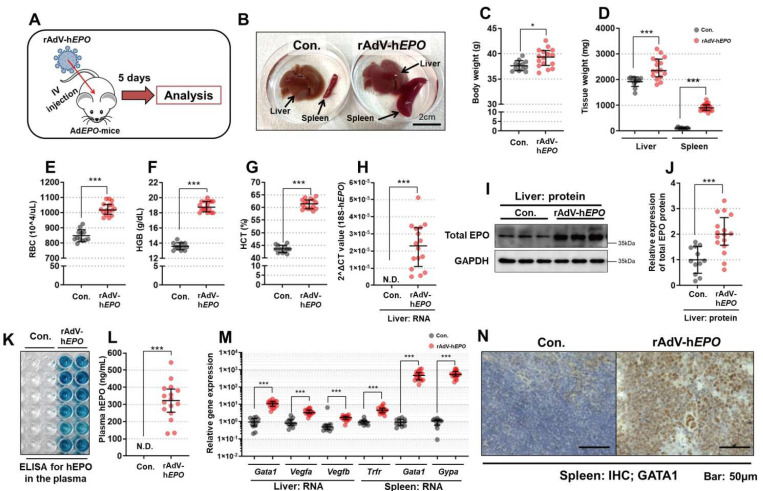
Establishment of a gene-doping model using rAdV-h*EPO*. The phenotypes and parameters related to hematopoiesis were assessed in the Con. (*n* = 12) and rAdV-hEPO (*n* = 15) groups. (**A**) An overview of the experiment, (**B**) a photograph of the liver and spleen of a representative mouse, (**C**) body weight of the mice, (**D**) liver and spleen weight, (**E**) RBC count (10^4^/μL), (**F**) Hgb level (g/dL), (**G**) HCT (%), (**H**) expression of the h*EPO* gene in the liver, (**I**) representative images of total EPO and GAPDH on WB analysis of liver specimens, (**J**) quantification of the band intensity on WB, (**K**) a representative image of the actual reaction plate of ELISA for the hEPO protein, which was performed as duplicate measurements of 6 samples, (**L**) results of quantification with ELISA, (**M**) analysis of the expression levels of genes encoding hematopoietic markers in the liver and spleen, (**N**) micrographs of GATA1-stained spleen sections evaluated using IHC. N.D.: not detected. * *p* < 0.05, *** *p* < 0.001.

The short-term experiments were conducted to establish a gene-doping mouse model with rAdV-h*EPO* and to develop methods to detect direct and indirect proof of gene doping. An overview of these experiments is shown in Figure 1A. After 1 week of acclimatization, mice were randomly assigned to the control (Con.) or rAdV-h*EPO* groups. The mice in the rAdV-h*EPO* group (*n* = 16; named Ad*EPO* mice) received injections of the AdV-h*EPO* vector (4.0 × 10^11^ vp/100 µL/mouse) into the orbital sinus under systemic isoflurane inhalation anesthesia. The mice of the Con. group (*n* = 12) received injections of the 10% glycerol/PBS (100 µL/mouse) buffer used to suspend the rAdV. Five days after the injection, whole blood was obtained with EDTA-2Na as an anticoagulant from the inferior vena cava under systemic isoflurane inhalation anesthesia, after which the mice were euthanized. The collected whole blood was subjected to preprocessing for further analysis. Samples of liver and spleen were also harvested and flash-frozen in liquid nitrogen until further analysis. Other samples of spleen tissues were immersed into 10% formalin neutral buffer solution overnight to obtain paraffin block specimens. Supplementary experiments were conducted using the rAdV-*ZsGreen1* (*ZSG*) vector to confirm the effects of h*EPO*-free rAdV on the phenotypes.

Long-term experiments were conducted to investigate how long direct and indirect proof of gene doping could be positively detected from approximately one drop of whole blood. An overview of these experiments is shown in Figure 5A. After 1 week of acclimatization, small whole-blood samples (approximately 100 µL; 2 drops) from mice (n = 12) were collected with EDTA-2Na using cuts made approximately 2 mm from the tail tip (Pre-time point). Then, the AdV-hEPO vector was injected using the methods described above. Subsequently, using the same method described above, blood samples were continually collected at the time points shown in Figure 5B–J until 30 days after the injection. In order to eliminate the adverse effects of continuous blood collection on mice (for example, anemia, hematopoietic stimulation, etc.) as much as possible, we selected a blood-collection method that only obtained small amounts (approximately 100 µL) from the tail tip. The collected blood samples were divided into two samples of approximately 50 µL each, and DNA and RNA were extracted from these samples and subjected to further analyses.

### 2.3. Measurements of General Hematopoietic Markers

Hematological indicators of RBCs, namely, the RBC count, hemoglobin (Hgb) level, and hematocrit (HCT) value, from the whole-blood samples obtained in the short-term experiments were measured on an automatic blood analyzer (Celltac α MEK6458; NIHON KODEN, Shinjuku, Tokyo, Japan) using 50 µL of whole blood.

### 2.4. Western Blotting

To confirm the expression of the hEPO protein in the liver in the short-term experiment, Western blotting (WB) was performed. To extract the total protein from the liver, liver specimens were homogenized in lysis buffer (50 mM Tris-HCl pH 7.4, 150 mM NaCl, 1% NP40, 1 mM EDTA) with a protease inhibitor cocktail (Cat# 25955-11; Nacalai Tesque, Nakagyo, Kyoto, Japan) using a bead crusher (TissueLyser LT, Cat# 85600; QIAGEN, Hilden, Germany). The homogenized liver lysate was then centrifuged at 12,000× *g* for 15 min, and the supernatant was collected. The concentration of total protein was measured using a BCA kit (Cat# T9300A; Takara Bio, Kusatsu, Shiga, Japan) and adjusted to 2 µg/mL. The supernatant was mixed with 2× loading buffer containing 2-mercaptoethanol and denatured at 95 °C for 5 min. Subsequently, 10 µL samples including 10 µg of protein were subjected to sodium dodecyl sulfate polyacrylamide gel electrophoresis (SDS-PAGE) using 15% gel at 140 V for 70 min, after which the separated proteins in the gel were transferred to a polyvinylidene fluoride (PVDF) membrane using the wet transfer method at 40 V overnight. This membrane was blocked with TBS-T buffer (50 mM Tris-HCl, 150 mM NaCl, 0.05% Tween-20) including 5% skim milk for 120 min and then washed with TBS-T buffer 3 times for 10 min. The membrane was incubated with the primary antibody for EPO (Cat# sc-80995; Santa Cruz Biotechnology, Dallas, TX, USA) at 100-fold overnight at 4 °C with gentle shaking and then washed with TBS-T buffer three times for 10 min. The washed membrane was incubated with anti-rabbit IgG HRP-linked antibody (Cat# 7074, 3000-fold; Cell Signaling Technology, Danvers, MA, USA) with gentle shaking for 30 min at room temperature. After washing the membranes three times for 10 min, the target protein bands were visualized with a chemiluminescence reagent (EzWestBlue; Cat# WSE-7140; ATTO, Taito, Tokyo, Japan) on ImageQuant LAS 4000 (Cytiva, Marlborough, MA, USA). The band images were exported as 16-bit TIFF images. The luminance of the bands of the TIFF images was quantified using ImageJ Fiji (ver. Java 8). The antibody for glyceraldehyde-3-phosphate dehydrogenase (GAPDH, Cat# 60004-1-Ig, 3000-fold; Proteintech, Rosemont, IL, USA) was also used as the loading control with anti-mouse IgG HRP-linked antibody (Cat# 7076, 3000-fold; Cell Signaling Technology, Danvers, MA, USA).

### 2.5. Enzyme-Linked Immunosorbent Assay

Enzyme-linked immunosorbent assay (ELISA) was performed to confirm the secretion of hEPO into the blood from the liver in Ad*EPO* mice in the short-term experiment. Anti-hEPO (Cat# 500-P318; PeproTech, Cranbury, NJ, USA) was diluted to 0.5 µg/mL in PBS as a capture antibody, and 100 µL of the antibody solution was then applied into ELISA plates to obtain duplicate measurements. The plate was incubated at 4 °C overnight and then washed with PBS containing 0.05% Tween 20 (PBS-T) four times; then, 100 µL of the plasma samples diluted 10-fold with PBS were added to the wells and incubated for 1 h at room temperature. Solutions of hEPO recombinant protein (Cat# 100-64; PeproTech) in PBS were also added to generate a standard hEPO curve between 100 ng/mL and 10 pg/mL. After incubation, the plate was washed in the same way as described previously, and a 100 µL solution of a detection antibody (biotinylated anti-hEPO; Cat# 500-P318BT; PeproTech) diluted with PBS-T was applied to the wells at a concentration of 0.25 ng/µL, followed by incubation for 1 h at room temperature. After incubation, the plate was washed in the same way as described previously, and a 100 µL solution of HRP-conjugated streptavidin (Cat# SA00001-0; Proteintech, Rosemont, IL, USA) was diluted 5000-fold with PBS-T was applied to the wells. The plate was incubated at room temperature for 30 min and then washed the same way as described previously. After washing, a 100 µL mixture of coloring reagent and substrate (ELISA POD Substrate A.B.T.S Kit, Cat# 14351-80; Nacalai Tesque, Nakagyo, Kyoto, Japan) was applied and incubated for 20 min, followed by application of 100 µL of stop reagent for the coloring reaction. Finally, the absorbance at 405 nm with a reference at 600 nm was measured using a microplate reader. Using the absorbance data, a standard curve as a 4-parameter logistic of the Rodbard was created in ImageJ Fiji (Life-Line version, updated on 30 May 2017), and the concentration of hEPO in the plasma was calculated in duplicate measurements based on the standard curve (R^2^ = 0.99).

### 2.6. TB Green qPCR Assay for Tissue RNAs

To confirm the expression of hematopoietic marker genes in the liver and spleen in the short-term experiment, the TB Green qPCR assay was performed as an intercalation method. RNA extraction from the liver and spleen was performed using RNAiso Plus (Cat# 9180; Takara Bio, Kusatsu, Shiga, Japan) according to the manufacturer’s instructions. The extracted RNA solution in Milli-Q Water (Merck Millipore, Burlington, MA, USA) was diluted and adjusted to a concentration of 100 ng/µL. Then, 500 ng of RNA was used to prepare cDNAs using PrimeScript RT Master Mix (Cat# RR036A; Takara Bio) according to the manufacturer’s instructions. The cDNAs were diluted 10× using Milli-Q Water and subjected to a quantitative real-time PCR (qPCR) assay based on the intercalator-fluorescence dye. The qPCR assay was performed to quantify the gene expression of hematopoietic markers in the liver and spleen by using TB Green Premix Ex Taq II (Cat# RR820; Takara Bio) with primers from QuantStudio 5 Real-Time PCR Systems (Thermo Fisher Scientific, Waltham, MA, USA) as duplicate measurements. The targeted gene list and primer sequences are shown in Supplementary Materials, Table S1. The template volume and primer concentrations were 2 µL and 100 nM, respectively, for a total reaction volume of 10 µL per well. Negative control wells were also established using pure water instead of the template. The conditions for thermal cycling were 95 °C for 5 min, followed by 40 cycles of 95 °C for 2 s and 60 °C for 20 s and a melt curve stage. Subsequently, the ΔΔCt method referencing *18s rRNA* was used to calculate the relative gene expression values.

### 2.7. Immunohistochemistry

Immunohistochemistry (IHC) was performed to confirm the expression and localization of GATA1, a common hematopoietic marker, in the short-term experiment. In this experiment, 4 µm sections of the spleen on slides were deparaffinized with Lemosol A (Cat# 126-04413; FUJIFILM Wako Pure Chemical Corporation, Osaka, Japan) and rehydrated using ethanol and running water. After 3 min of rinsing with running water, the endogenous peroxidase in the tissues was removed by treatment with 3% hydrogen peroxide/methanol for 10 min. After washing with PBS for 1 min with gentle shaking, the sections were placed in 0.01 M citrate buffer and subjected to antigen activation at 121 °C for 10 min. Then, the sections were gently washed with PBS and blocked with 5% bovine serum albumin (BSA)/PBS-T for 1 h at room temperature. The primary antibody for GATA1 (Cat# 10917-AP; Proteintech, Rosemont, IL, USA), diluted 100-fold with 1% BSA/PBS-T, was applied to the sections and incubated at 4 °C overnight. On the next day, after washing the slides with PBS-T three times for 15 min, anti-rabbit IgG HRP-linked antibody (Cat# 7074; Cell Signaling Technology, Denvers, MA, USA) diluted 1:100 with 1% BSA/PBS-T was applied to the sections and incubated for 1 h at room temperature after washing with PBS. Subsequently, 3,3-diaminobenzidine tetrahydrochloride (DAB) solution in the peroxidase stain DAB Kit (Cat# 2598-50; Nacalai Tesque, Nakagyo, Kyoto, Japan) was applied to the section, and the section was incubated for 5 min at room temperature, followed by washing with PBS and counterstaining with hematoxylin. The sections were then dehydrated and mounted using Malinol (Cat# 2009-3; MUTO PURE CHEMICALS, Bunkyo, Tokyo, Japan) and observed under a microscope (BZ-X710; Keyence, Osaka, Japan).

### 2.8. DNA Extraction from Whole-Blood Samples

A phenol/chloroform/isoamyl alcohol solution (Cat# 25970-56; Nacalai Tesque) was used to extract total DNA from 100 µL (short-term experiment) or 50 µL (long-term experiment) of whole blood in accordance with the manufacturer’s instructions. The pellets of DNA were dissolved in 50 µL of Milli-Q Water, and the DNA solutions were subjected to the TaqMan qPCR assay to obtain direct proof of gene doping.

### 2.9. TaqMan qPCR Assay

**Figure 2:**
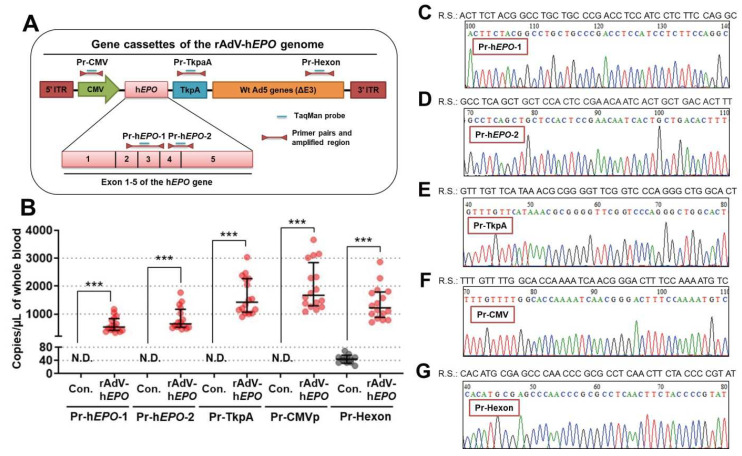
Direct proof in whole-blood DNA from AdEPO mice. (**A**) Strategy for designing specific primer-probe pairs for the viral genome. “Pr” indicates a primer-probe pair. (**B**) Detection of direct proof using the TaqMan qPCR assay performed with whole-blood DNA from the control (*n* = 12) and AdEPO mice (*n* = 15). (**C**–**G**) Sanger sequence analysis of the pooled amplicon on the TaqMan qPCR assay using five primer-probe pairs; R.S. indicates a reference sequence that completely matches the amplicon over 40 nucleotides on any primer-probe pair. N.D.: not detected. *** *p* < 0.001.

Our previous studies have shown that whole-blood DNA contains high levels of viral genome DNA fragments as direct proof [5,6,7]; therefore, we performed the TaqMan qPCR assay with whole-blood DNA. For detection of direct proof of gene doping in whole-blood DNA in the short- and long-term experiments using the TaqMan qPCR assays, the primers and TaqMan probes were designed to target the *EPO* gene (2 types), TkpA (thymidine kinase polyA from herpes simplex virus), hexon (major virus capsid protein from adenovirus), and CMVp (cytomegalovirus promoter) to ensure specific amplification of the rAdV-h*EPO* genome using Primer-BLAST (NIH National Library of Medicine, Bethesda, MD, USA). The primers and TaqMan probes for the two types of h*EPO* genes were prepared with exon-exon junctions (exons 2–4 and 4–5), which is a nonamplifying structure in the human genome [6,24,25]. An overview of these design strategies is shown in Figure 2A. The primers and probes were also checked for specificity with in silico PCR using Primer-BLAST; these evaluations confirmed the absence of amplification in the human and mouse genomes. The sequences of the primers and TaqMan probes are shown in Supplementary Table S1. The primers and probes in a double quencher system were systemized by Integrated DNA Technologies (Coralville, IA, USA). Next, the TaqMan qPCR assay was performed in duplicate to detect direct proof in whole-blood DNA by absolute quantification using PrimeTime Gene Expression Master Mix (Cat# 1055771; Integrated DNA Technologies) with the primers and TaqMan probes on QuantStudio 5 Real-Time PCR Systems (Thermo Fisher Scientific, Waltham, MA, USA). The template DNA volume was 2 µL, and the primer and probe concentrations were 200 nM and 100 nM, respectively, for a total reaction volume of 10 µL per well. Negative control wells were also established using pure water instead of a template. ph*EPO*-Myc-DDK-tags including the h*EPO* gene or pAd/CMV/V5-DEST including TkpA, hexon, and CMVp were used at 100 pg/μL to prepare a standard curve for absolute quantification, and the range of the standard curve was set to 1.19–19 × 10^7^ copies/μL for the ph*EPO*-Myc-DDK-tag and 2.53–10.1 × 10^6^ copies/μL for the pAd/CMV/V5-DEST. The conditions of thermal cycling for all primer-probe pairs were 95 °C for 5 min, followed by 40 cycles of 95 °C for 2 s and 60 °C for 20 s. All standard curves had R^2^ > 0.98.

### 2.10. Sanger Sequencing

After the TaqMan qPCR, solutions including the amplicon were pooled in a 1.5 mL microtube and then subjected to electrophoresis using a 2% agarose gel. The bands of the DNA amplicons were visualized using ethidium bromide solution. Next, the bands of desired fragment sizes were confirmed, indicating that specific amplifications of all primer-probe pairs were successful in the qPCR assay (Supplementary Figure S7). Subsequently, the gels, including the bands, were cut and purified using a NucleoSpin Gel and PCR Clean-up kit (Cat# 740609; Takara Bio, Kusatsu, Shiga, Japan). Next, to check the sequence of the DNA amplicons, 5 ng of the purified DNA was subjected to Sanger sequencing via outsourcing to an external company (GENEWIZ, Shinagawa, Tokyo, Japan). The Sanger sequencing data were analyzed with CLC Sequence Viewer ver. 8.0 (QIAGEN, Hilden, Germany) and BioEdit ver. 7.2.5 (developer: Tom Hall, Carlsbad, CA, USA).

### 2.11. Total RNA-Seq

Total RNA-seq was performed to identify genes as a novel form of indirect proof based on the concept of the ABP. Total RNA of the mice in the short-term experiments was extracted from 100 µL of whole blood using RNAiso Blood (Cat# 9112; Takara Bio) according to the manufacturer’s instructions. The RNA pellets were dissolved in 30 µL of Milli-Q Water, and the RNA solutions of eight samples (Con.: *n* = 4 and rAdV-h*EPO*: *n* = 4) were checked for integrity using Agilent RNA 600 Nano Kit (Cat# 5067-1511; Agilent Technologies, Santa Clara, CA, USA) on a Bioanalyzer (Agilent Technologies). The RNA Integrity Number (RIN) of all samples was 8.8 or higher; thus, the RNAs of all eight samples could be subjected to library preparations for total RNA-seq. Using 300 ng of the RNAs from each sample, libraries were created using the NEBNext Ultra II RNA Library Prep Kit for Illumina and the NEBNext rRNA Depletion Kit v2 (Cat# E7770S and E7400L; New England Biolabs, Ipswich, MA, USA) according to the manufacturer’s instructions, and the final PCR cycle was 12. Concentrations and size distributions of the libraries were measured using an Agilent DNA 7500 kit (Cat#5067-1506; Agilent Technologies, Santa Clara, CA, USA) with Bioanalyzer. All samples were passed for analyses on NGS equipment.

The libraries were pooled, and the concentrations were adjusted to 1 nM. The pooled libraries were subjected to denaturation and neutralization. Subsequently, the libraries were diluted to 1.8 pM and then applied for an NGS run using NextSeq500/550 v2.5 (75 Cycles) Kits (Cat#20024906; Illumina, San Diego, CA, USA) in the NextSeq 500 System (Illumina). The sequencing was performed with paired-end reads of 36 bases. After the sequencing run, FASTQ files were exported, and the basic information of the NGS run data were checked on CLC Genomics Workbench 20.0.3 software (QIAGEN, Hilden, Germany). In the quality assessment of the reads, a PHRED score over 20 was confirmed for 99.73% of all reads, indicating the success of the run. The read number was approximately 37 to 45 million per sample as paired-end reads.

### 2.12. Bioinformatics Analysis

The following analysis was performed to identify genes as novel indirect proof based on the concept of the ABP using NGS run data. FASTQ files were mapped to the mouse genome (mm 10) using the CLC Genomics Workbench software (QIAGEN). A statistical differential expression test was performed by empirical analysis using the Differential Expression in Two Groups tool in the software. Differentially expressed genes (DEGs) defined by the cutoff levels were considered with a false discovery rate (FDR) < 0.01 and a 3-fold change cutoff. A principal component analysis (PCA) plot was created using the CLC software. Transcripts Per Kilobase Million (TPM) was used as an expression value for figure visualizations. The genes with low read counts were filtered by 0 in all samples. Gene Set Enrichment Analysis (GSEA) software [26] was used to perform the enrichment analysis. Expression data sets and phenotype files were created and imported into GSEA software. On the basis of “Using RNA-seq Datasets with GSEA”, input normalized data were created with DEseq2 using TCC-GUI to perform GSEA [27]. The GSEA process was performed with the default settings and the h.all.v7.4.symbols, C2.cgp.v7.4.symbols, C2.cp.v7.4.symbols, and C5.go.v7.4.symbols data sets. Cell-type-specific analysis (CTSA) was performed using the TissueEnrich R package with the default settings [28] with RNA single-cell-type data from The Human Protein Atlas (https://www.proteinatlas.org/ (accessed on 30 March 2021)) to calculate cell-type-specific gene enrichment. The names of the DEGs were matched to the corresponding human gene names with the biomaRt R package for conducting CTSA [29]. Tissue-specific expression analysis (TSEA) was performed using the TissueEnrich web tool (https://tissueenrich.gdcb.iastate.edu/ (accessed on 30 March 2021)) [28] with the Mouse ENCODE Dataset to calculate tissue-specific gene enrichment. The Database for Annotation, Visualization and Integrated Discovery (DAVID version 6.8, LHRI) web tool was used to elucidate the enrichment terms with default settings [30].

### 2.13. TB Green qPCR Assay for Whole-Blood RNA

To confirm whether the genes identified as indirect proof of gene doping showed reproducibility for the total RNA-seq results, a TB Green qPCR assay was performed for all samples (Con.: *n* = 12, rAdV-h*EPO*: *n* = 15) in the short-term experiment. RNA extraction from 100 µL whole-blood samples was performed using the same methods mentioned in the “Total RNA-seq” section. Subsequently, the TB Green qPCR assay for the identified genes as indirect proof was performed using the same method described in the “TB Green qPCR assay for tissue RNAs” section. The primer sequences used in this section are shown in Supplementary Table S1.

In addition, to investigate the period for which the identified indirect proof could be positively detected, the same qPCR assay was also performed using 50 µL whole-blood samples (*n* = 12) from the long-term experiment. In this experiment, cDNA concentrations were additionally measured using the QuantiFluor ssDNA System (Cat# E3190; Promega, Madison, WI, USA) according to the manufacturer’s instructions. Subsequently, the expression values of each targeted gene were normalized by their cDNA concentrations because the expression levels of the normalizer gene (*18s rRNA*) significantly changed according to the passage of days. In the actual experiment, we also considered the use of other normalizer genes. *Gapdh* and *Rpl13a* also showed significant changes upon rAdV injection. For this reason, we chose to normalize gene expression values to the total amount of cDNA.

### 2.14. Statistical Analysis

All data except the total RNA sequence data were statistically analyzed using GraphPad Prism version 9.0.2 (GraphPad, San Diego, CA, USA). All experimental data were first evaluated with the Shapiro–Wilk normality test to check the normality of the distributions. Subsequently, nonparametric tests were used for all data. For comparisons of two groups, the Mann–Whitney U test was performed. Comparisons of three or more groups were performed with Kruskal–Wallis H tests (one-way ANOVA of ranks) followed by a two-stage Benjamini, Krieger, and Yekutieli FDR procedure as a post-hoc test. A *p*-value less than 0.05 was considered to indicate statistical significance. All graphs without data from the bioinformatics analysis of the total RNA-seq are shown as individual plots and medians with interquartile ranges.

## 3. Results

### 3.1. Mouse Model of Gene Doping Established Using rAdV-hEPO

Figure 1A shows an overview of this experiment. A phenotypic analysis performed 5 days after the injection of rAdV-h*EPO* showed a significant increase in the body weight of Ad*EPO* mice in comparison with the control mice. The livers and spleens of Ad*EPO* mice also showed significant hypertrophy (Figure 1B–D). The RBC counts, Hgb levels, and HCT values, which served as hematological markers in whole blood, were also significantly increased (Figure 1E–H), indicating the occurrence of hematopoiesis.

To confirm whether hEPO was expressed and secreted in/from the liver, qPCR, WB, and ELISA were performed using liver RNA, protein lysate, and plasma, respectively. The results showed that the total EPO protein expression was significantly elevated in Ad*EPO* mice, and the hEPO hormone was detected in the plasma only in Ad*EPO* mice along with positive expression of the h*EPO* gene in the liver (Figure 1H–L). The raw data from the WB analysis are shown in Supplementary Figures S1 and S2.

In addition, we confirmed whether the gene expression levels of the downstream targets of EPO in hematopoiesis signaling had changed in the livers and spleens of Ad*EPO* mice. The results showed significantly upregulated expression levels of *Gata1*, *Vegfa*, and *Vegfb* in the livers of Ad*EPO* mice. Moreover, the expression levels of *Gata1*, *Trfr*, and *Gypa* were drastically upregulated in the spleens of Ad*EPO* mice (Figure 1M). In the spleen, increased GATA1 protein expression was also confirmed using IHC. These results suggested that gene delivery to the liver, secretion of hEPO into blood, and hematopoiesis in the spleen were successfully achieved by injection of rAdV-h*EPO*, indicating the hematopoietic function of hEPO.

To investigate the effects of h*EPO*-free rAdV on these phenotypes and gene expression, we conducted a supplementary experiment using rAdV-*ZSG* as a form of h*EPO*-free rAdV that could produce green fluorescent protein. Induction of rAdV-ZSG did not yield phenotypes and gene expression related to hematopoiesis (Supplementary Figure S3A–I). Thus, rAdV alone had no hematopoietic effect in mice. Overall, these results clearly indicated the establishment of a gene-doping model with rAdV-h*EPO* in this study.

### 3.2. Direct Proof of Gene Doping in Whole-Blood DNA from AdEPO Mice

Using specific primer-probe pairs (Figure 2A) and whole-blood DNA in the TaqMan qPCR assay, the results provided direct proof of gene doping with statistical significance for all five primer-probe pairs (Figure 2B). The median copy numbers of the targeted DNA fragments were confirmed to be approximately 500–1500 copies/µL in whole blood. However, positive detection was also confirmed in the control mice when using the primer-probe pairs to detect the hexon region (Figure 2B; Pr-hexon).

The DNA sequences of the amplicon for each TaqMan qPCR assay were confirmed using pooled amplicon samples with gel extraction using the Sanger sequencing method. The results showed correct sequences over 40 nucleotides in any primer-probe pair (Figure 2C–G). Therefore, these results suggested that these primer-probe pairs can accurately detect direct proof in Ad*EPO* mice as a gene-doping model. On the other hand, the possibility of experimental contaminations of DNA fragments of the hexon region could not be ruled out.

### 3.3. RNAs as Novel Indirect Proof of Gene Doping in Whole-Blood RNA

**Figure 3:**
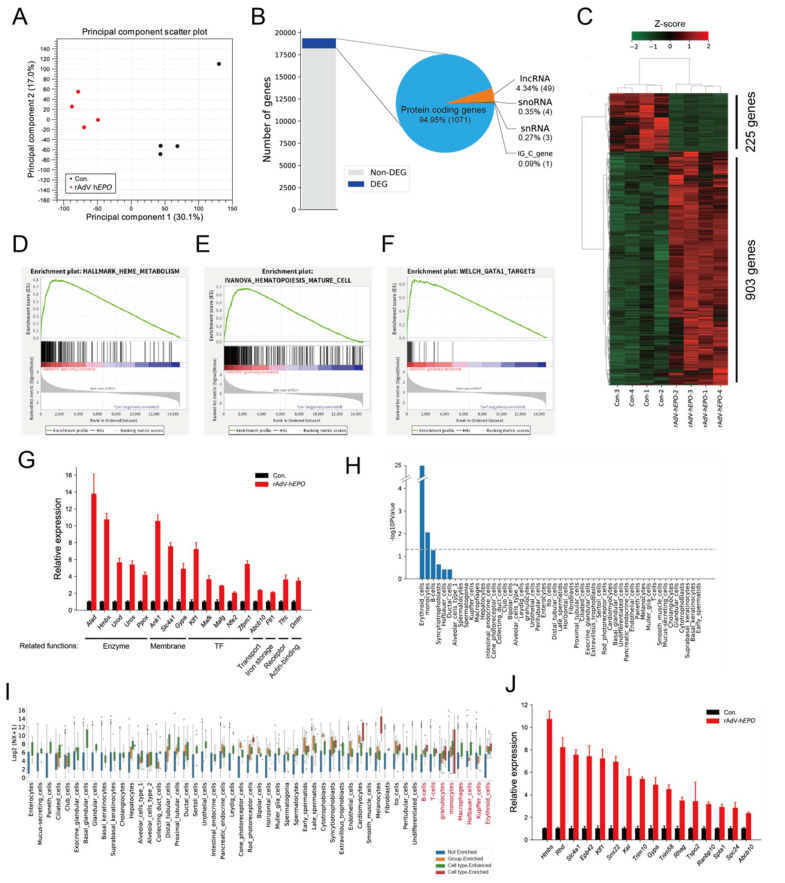
Hematopoietic changes caused by rAdV-h*EPO* supporting the whole-blood RNA-seq findings. (**A**) PCA plot showing similarities between samples. (**B**) Bar plot and pie chart showing the 1128 DEGs included among all detected genes in whole blood and rates of the gene types. (**C**) Heat map indicating the DEG profile. Values in the rows are z-scores. GSEA showing enriched terms: heme metabolism (**D**), hematopoiesis mature cell (**E**), and GATA1 target (**F**) terms. (**G**) Relative gene expression levels (Con. = 1.0) showing gene expression in the GATA1 target term in (**F**). Biological-related functions are described under gene names. The genes were visualized according to the GSEA enrichment results with a “Yes” value. Error bars indicate the standard error of the mean (SEM). (**H**) Bar plot showing the CTSA to predict cell-type-specific enrichment in DEGs. The gray line represents a significant threshold (*p* < 0.05). (**I**) Box plot showing single-cell gene normalized expression (“NX”) of DEGs in each cell type. Single-cell gene expression levels of DEGs were classified by cell-type-specific definitions by TissueEnrich for each cell-type. (**J**) Relative gene expression levels (Con. = 1.0) showing erythroid cell-enriched gene expressions in DEGs. Abbreviations: long non-coding RNA (lncRNA), small nucleolar RNA (snoRNA), small nuclear RNA (snRNA), and immunoglobulin constant germline gene (IGC gene).

Total RNA-seq and bioinformatics analysis were performed to identify potential indirect proof of gene doping using whole-blood RNA. The PCA plot showed that the comprehensive gene expression profiles considerably differed between control and rAdV-h*EPO* mice (Figure 3A). Gene expression profiles were relatively similar across the samples within each group, except for one control sample (Figure 3A). The statistical analysis identified 1128 DEGs (Figure 3B). These DEGs included protein-coding genes (94.95%), long non-coding RNA (lncRNA) genes (4.34%), small nucleolar RNA (snoRNA) genes (0.35%), small nuclear RNA (snRNA) genes (0.27%), and immunoglobulin constant germline (IGC) genes (0.09%). The gene expression profile included 903 upregulated and 225 downregulated genes (Figure 3C). Interestingly, GSEA predicted several gene sets associated with hematopoiesis-related terms (Figure 3D–F). In particular, the genes included in the GATA1 target term are known to be related to RBC development and component functions (Figure 3G). In addition, GSEA indicated that genes for specific elements of hemopoiesis-related mechanisms were significantly enriched, such as the mitochondria-related electron transport chain, OXPHOS pathways, and metal ion homeostasis-related terms (Supplementary Figure S4). Next, we hypothesized that the DEGs might include RBC-specific genes that could be candidate genes for gene doping with rAdV-h*EPO*. Thus, CTSA was performed to identify potential cell-type-specific genes in circulating RNA. Erythroid cell-specific genes were most significantly enriched on CTSA (Figure 3H). In addition, DEGs were confirmed to have other blood cell-type-specific gene changes, although those cell types were not enriched on CTSA (Figure 3I). Thus, these blood cell-specific genes, particularly RBC-specific genes, may serve as indirect proof of rAdV-h*EPO* gene doping by providing supporting evidence for gene doping (Figure 3J).

Furthermore, to identify the potential genes in whole blood that may reflect tissue status, we conducted TSEA, which showed that E14.5-liver-, bone marrow-, and spleen-categorized genes were enriched (Supplementary Figure S5A). In addition, we noticed that the E14.5-liver-categorized genes overlapped with the bone marrow-categorized genes (Supplementary Figure S5B,C). These 42 overlapping genes represented enriched terms such as porphyrin-containing compound biosynthetic process, erythrocyte development, protoporphyrinogen IX biosynthetic process, heme biosynthetic process, and erythrocyte differentiation (Supplementary Figure S5D). For example, *Rhd*, *Kif1*, *Rhag*, and *Gata1* were included in the erythrocyte development term (Supplementary Figure S5E). In particular, Gata1 was upregulated in whole blood (Supplementary Figure S5F), consistent with the results for tissues (Figure 1M). These results indicate a hematopoietic status inside tissues after rAdV-h*EPO* injection, consistent with the tissue phenotype. In addition, the changes in the overall RNA expression profiles of these hematopoiesis-associated genes may serve as indirect proof of gene doping based on the concept of the ABP.

**Figure 4:**
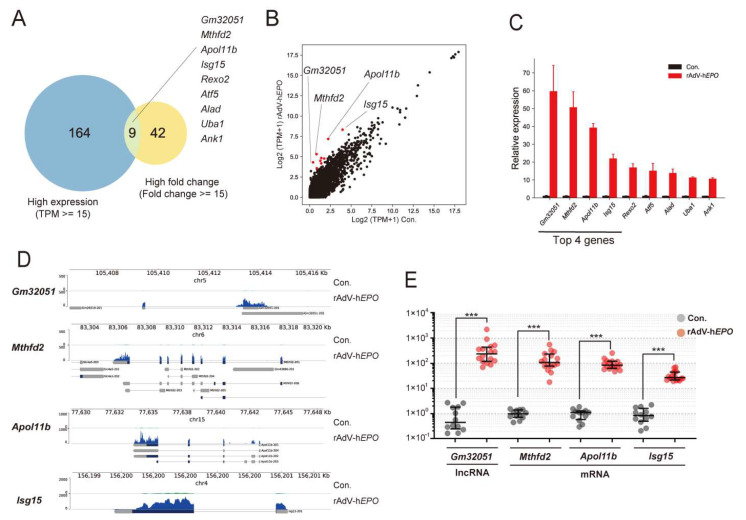
Single RNAs identified as indirect proof by bioinformatics screening. (**A**) Venn diagram showing the number of DEGs with filtering for high expression and high fold expression. (**B**) Scatter plot showing nine overlapping genes with filtering. (**C**) Relative gene expressions (Con. = 1.0) showing nine overlapping genes. (**D**) Genome browser indicating the read mapping results of the top four genes. (**E**) Confirmation of the top four genes as indirect proof by using the TB Green qPCR assay. *** *p* < 0.001.

Next, we screened the single candidate genes that showed drastic changes in expression upon gene doping. These genes were identified by filtering high fold changes and high expression levels (Figure 4A) because the gene should be measurable with high sensitivity on qPCR. Nine candidate genes were screened in this assessment: *Gm32051*, *Mthfd2*, *Apol11b*, *Isg15*, *Rexo2*, *Atf5*, *Alad*, *Uba1*, and *Ank1* (Figure 4A). Among these, we selected the top four genes: *Gm32051*, *Mthfd2*, *Apol11b*, and *Isg15* (in descending order of expression fluctuations) (Figure 4B,C). In addition, we confirmed that the coverage graphs provided accurate read mappings (Figure 4D). The four identified genes were quantified using the TB Green qPCR assay to determine the reproducibility of the total RNA-seq data. The results yielded similar quantitative values for the total RNA-seq data, with the expression values of the identified RNAs being more than 30–100-fold in Ad*EPO* mice in comparison with control mice (Figure 4E). Surprisingly, since these screened genes involved functions unrelated to *EPO*, these results suggest that even a single gene of the identified RNAs can be indirect proof of gene doping based on the concept of the ABP.

### 3.4. Direct and Indirect Proof Were Detected for Up to Approximately 30 Days Using DNA/RNA from a Drop of Whole Blood

**Figure 5:**
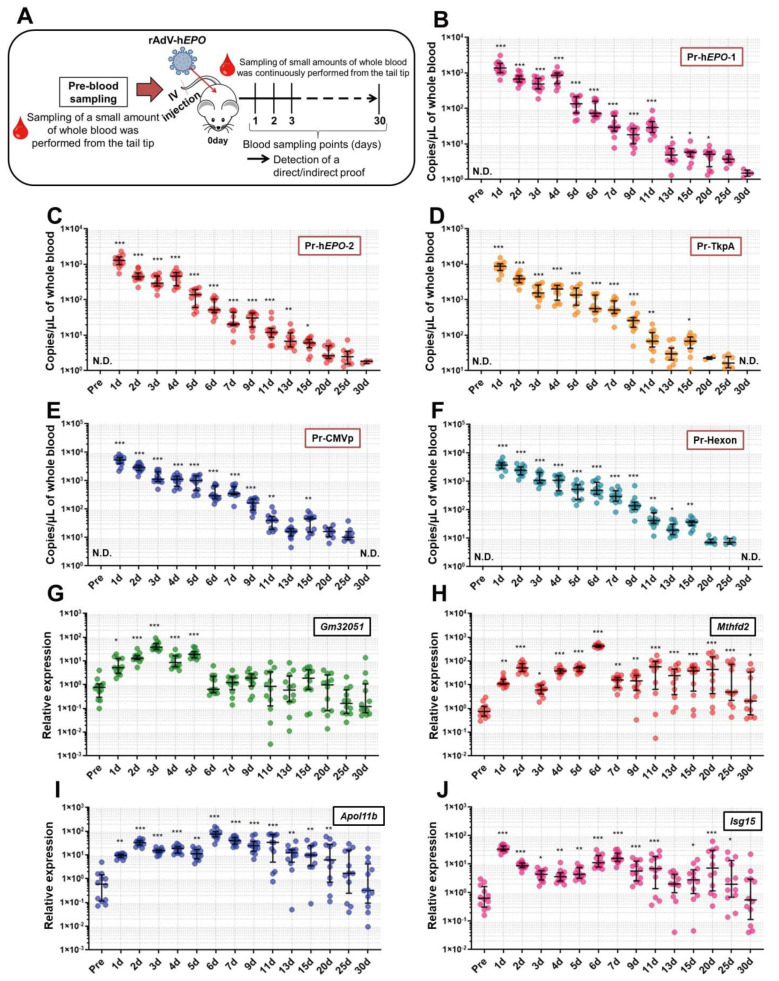
Detection of direct and indirect proof for a long time period. To detect direct and indirect proof, small amounts of whole blood were harvested from the tail tips of mice (*n* = 12) until 30 days after the injection. From approximately 50 μL of whole blood, DNA/RNA was extracted, and the TaqMan or TB Green qPCR assays were performed to detect direct and indirect proof. (**A**) Overview of the experiments in this section, (**B**–**F**) continuous detection of direct proof for 30 days by five primer-probe pairs using the TaqMan qPCR assay; “Pr” refers to a primer-probe, (**G**–**J**): detection of changes in gene expression as indirect proof using the TB Green qPCR assay. N.D.: not detected. * *p* < 0.05, ** *p* < 0.01 and *** *p* < 0.001 vs. pre-value.

Figure 5A shows an overview of the experiments performed in this section. The results showed that direct proof of viral genome DNA was significantly detected until 15 to 20 days after the injection (Figure 5B–F). Furthermore, several mice qualitatively showed the target DNA fragments for the h*EPO*-1 and -2 primer-probe pairs (Pr-h*EPO*-1 and -2; Figure 5B,C) until 30 days. Moreover, indirect proof of RNA expression levels identified from the total RNA-seq showed significant fluctuations, i.e., until only 5 days for Gm32051 as lncRNA and 20–30 days for other mRNAs (Figure 5G–J). The longest time of significant changes was observed for Mthfd2 (Figure 5H). These results indicate that direct proof of gene doping can be detected for a long time in association with changes in RNA expression as indirect proof.

## 4. Discussion

Despite the WADA ban on gene doping [3], the potential for *EPO*-based gene doping remains high because of the rapid advancements in gene therapy. To date, several studies have developed protocols for detecting doping with the *EPO* gene [6,18,19,24,25]. Moreover, the WADA has recently established laboratory guidelines for PCR methods in gene-doping tests [11]. Thus, large-scale gene-doping testing will be required in the future. However, despite providing detailed descriptions of validation methods for PCR analyses, the WADA guidelines [11] include no description of detection methods for individual target genes or vectors. Moreover, animal models of gene doping using AdV-h*EPO* have not been established, and no previous report has described a gene-doping test based on AdV-h*EPO*. Therefore, in this study, we first attempted to develop a mouse model of gene doping using an rAdV-hEPO vector, which will facilitate the development of detection methods for individual target genes and vector-genome sequences.

The gene and protein expression of hEPO in the liver was confirmed in Ad*EPO* mice, indicating that the h*EPO* gene was successfully delivered to the liver. Moreover, secretion of hEPO protein as a hormone into blood was also confirmed in Ad*EPO* mice. The expression of *Gata1* (regulator of hematopoietic signaling) [31,32], *Gypa*, and *Trfr* (specific markers of RBCs) [33,34,35] were drastically increased in the spleens of Ad*EPO* mice. Furthermore, the RBC count, Hgb level, and HCT were increased in Ad*EPO* mice. We also supplementally clarified that these effects were not the result of injecting the rAdV-*ZSG* vector as h*EPO*-free rAdV. These results indicate that the mouse model of gene doping made with the rAdV-h*EPO* vector was successfully established in this study. Moreover, the model may be useful to establish detection methods for gene doping.

Next, we aimed to develop detection methods for direct proof of gene doping by using the TaqMan qPCR assay. The primers were designed to include the exon-exon junction of the h*EPO* gene, which can specifically detect transferred genes [6,24,25] because if the exogenous gene is transferred to organs, the intron DNA sequences are not included. To detect robust proof of gene doping in mice, we also designed more primer-probe pairs to target DNA sequences of CMVp (cytomegalovirus promoter), hexon (major virus capsid protein from adenovirus), and TKpA (thymidine kinase polyA from herpes simplex virus), which are included in the rAdV-genome DNA sequence. These sequences are well used in genetic engineering experiments, and they are also present in wild herpes simplex viruses, adenoviruses, and cytomegaloviruses. Therefore, we thought that detection of these three DNA sequences in addition to the h*EPO* gene, including the exon-exon junction, may be stronger evidence than single detection. The results showed that all of the targeted DNA fragments could be detected, and the amplicons also had accurate DNA sequences as determined by Sanger sequencing. Therefore, these primer-probe pairs and qPCR conditions can be directly used to examine whole-blood DNA from athletes. Moreover, detection of multiple DNA fragments, not just a single DNA fragment, would provide robust scientific evidence for identifying the type of viral vector and for doping trials. A wide variety of vectors can be used for gene doping, but this study highlights the possibility of estimating the vector type used for gene doping by detecting a specific sequence of the viral vector. This finding could provide strong evidence for potential doping. However, some caution is required while interpreting these findings. The gene cassettes of CMVp, hexon, and Tkpa are also applied in vectors for gene therapy [36]. Moreover, recently, a vaccine for SARS-CoV-2 named ChAdOx1 nCoV-19 (AZD1222) may also include these gene cassettes because the vaccine was made using a replication-deficient simian adenoviral vector [37,38]. Therefore, individuals who have been treated with gene therapy or the vaccine may show false-positive test results, and determination of the individual’s history before conducting gene-doping tests is necessary. In particular, if gene-doping tests are performed at the 2020 Tokyo Olympics (held in July 2021), laboratories would need to confirm vaccination for SARS-CoV-2 in athletes because athletes are more likely to travel to Japan after being vaccinated because of the recent significant spread of COVID-19 worldwide.

To investigate the period for which genome-specific DNA fragments of the rAdV-h*EPO* vector can be detected as proof of gene doping, the TaqMan qPCR assay was performed using DNA obtained from single-drop blood samples chronologically harvested from the mouse model. The statistical analyses showed that significant proof of gene doping could be detected until 20 days after injection of the vector. In addition, several mice showed positive results for viral fragments until 30 days, which can be used as the basis for a qualitative test. The metabolic rate of mice has been shown to be several times faster than that of humans [39]. Hence, in humans performing gene doping with the rAdV-h*EPO* vector, proof of doping may be detected in the blood for over a month, a longer time than in mice. Our previous study using droplet digital PCR reported that when the DNA extracted from one drop of whole blood from an rAdV gene-doping mouse model was diluted and adjusted to a concentration of 10 ng/uL, significant positive proof of gene doping was detected until 5 days after the injection [5]. However, the present study showed that the use of an undiluted DNA solution could provide significant proof of gene doping for a longer time than that reported previously. These results suggest that undiluted DNA can be used to improve the sensitivity of detection, which is consistent with the WADA guidelines for PCR methods in gene-doping tests [11]. Therefore, we also propose the use of undiluted DNA from whole-blood samples in gene-doping tests for humans.

As mentioned previously, proof of gene doping was detected using one drop of whole-blood DNA. At present, a small examination device for point-of-care testing (POCT) is frequently used with human blood samples to examine several diseases at clinical sites [40]. With advancements in the technology underlying the current POCT devices, it may be possible to easily perform gene-doping tests with PCR using one drop of blood collected from the fingertip after puncture with a small needle. Therefore, we have begun to develop a small examination device that can be used for minimally invasive testing for gene doping on the sports field. However, there are major barriers to the development of such a device. Hgb and IgG have been reported to greatly inhibit PCRs [41]. In addition, large amounts of protein may also decrease the sensitivity of PCRs. To develop an immediate POCT device for use in the sports field, it is necessary to resolve these issues, and we have been performing repeated trial-and-error testing in this regard. Another possibility is to consider the use of dried blood spots (DBS) for gene-doping tests. In a recent report by Marchand et al. [42], only 20 μL of blood with plasmid including the *EPO* gene as a spike was applied as DBS, and a qPCR assay using the DBS detected approximately 1000 copies of the *EPO* gene fragments in the DNA. Moreover, no decrease in sensitivity was observed after storing the DBS at room temperature for one month. Since gene-doping tests using DBS offer multiple benefits, including simplification of sample collection, reduction in the burden on athletes, stabilization and reanalyzability of samples, etc., they may gain popularity in the future. Therefore, our next task is to collect a small amount of blood from the gene-doping model mouse (Ad*EPO* mice) as DBS and confirm the test sensitivity.

The ABP advocated by the WADA involves constant monitoring of selected biological variables that can indirectly reveal the effects of doping rather than attempting to detect the doping agent or method itself [20]. In other words, this concept is an attempt to detect traces of doping activity. The parameters evaluated as a part of ABP testing include general hematological parameters and steroid levels [20]. However, it is unclear whether RNA expression patterns in whole blood could be used as parameters for the ABP. Here, we considered that total RNA-seq technology can be applied to identify RNAs as a novel form of indirect proof based on the concept of the ABP because the peripheral blood transcriptome may dynamically reflect the biological mechanisms associated with increased EPO secretion. Our results identified 1128 DEGs in whole blood, and most of them were upregulated in Ad*EPO* mice (Figure 3B,C). The hematopoiesis-related terms such as GATA1 targets were significantly enriched in this profile by GSEA (Figure 3D–F). These genes, which encoded molecules such as erythrocyte surface proteins and erythroid transcription factors, are involved in erythropoiesis (Figure 3G) [43,44,45]. For example, *Tfrc* encodes the transferrin receptor protein 1 (sTfR), which is used in a diagnostic test for detecting rEPO hormone misuse by athletes [46]. In addition, we predicted that RBC-specific genes were significantly enriched (Figure 3H) and that other blood cell-type-specific genes also were contained in DEGs (Figure 3I). These results suggest that the expression changes, especially those in erythrocyte-specific genes, could reflect the hematopoietic effect of rAdV-h*EPO* and may serve as indirect proof based on the concept of the ABP. The enrichment analysis also showed that the erythropoiesis-related terms, such as mitochondria- and metal ion homeostasis-related terms, were significantly enriched in the upregulated genes (Supplementary Figure S4A,C,E,G). For example, the deletion of Rb caused moderate anemia, induced inhibition of mitochondrial biogenesis, decreased OXPHOS pathway activity and disturbed heme production and iron transport [47,48]. Thus, these results suggest that the blood transcriptome could explain and mirror the detailed hemopoietic mechanisms induced by rAdV-h*EPO* injection.

We also performed TSEA to predict the tissue status using DEGs because the blood transcriptome and protein expression patterns can partly predict tissue status [49,50,51]. TSEA showed enrichment of hematopoiesis-associated tissues such as E14.5 liver, bone marrow, and spleen (Supplementary Figure S5A). We noticed that the 42 predicted E14.5 liver- and bone marrow-specific genes overlapped with each other and that these genes were associated with enriched hematopoiesis-related terms, such as porphyrin-containing compound biosynthetic process and erythrocyte development (Supplementary Figure S5B–D). In particular, *Gata1*, known as the transcription factor for erythrocyte differentiation, was upregulated in blood, consistent with the findings for tissues (Supplementary Figure S5E,F). These results suggested that the whole-blood RNA profile may dynamically reflect the hematopoietic status in tissues injected with rAdV-h*EPO*.

Considering the results obtained to date, we believe that tracking significant changes in the overall profile of whole-blood RNA expression can serve as an ABP-based measurement approach since this idea is consistent with the concept of the ABP. Therefore, we recommend that total RNA-seq be employed in the generation of ABPs for gene doping in the future. However, to achieve this idea, it is essential to unify and establish a standardized workflow that includes RNA extraction, library preparation, NGS runs, and bioinformatics analyses, thereby yielding a robust protocol with a low scope for artificial contamination during experimental operations.

Next, to identify single RNA as novel indirect proof based on the concept of the ABP, in the gene-doping model, we screened potential single RNAs that showed high fold changes and high expression levels in the profile because the ideal genes should be measurable with high-sensitivity qPCR. Interestingly, to our knowledge, the screened genes are not known to be directly associated with EPO function. In particular, we identified several RNAs whose expression drastically increased by 80- to 100-fold or more upon injection of rAdV-h*EPO*. The identified RNAs were both mRNAs and lncRNAs for genes such as *Apol11b*, *Mthfd2*, *Gm32051*, and *Isg15* (Figure 4). *Apol11b* (*A330102K04Rik*) is reported to share homology with the apolipoprotein L family of proteins in humans, indicating its involvement in alterations in the lipid composition of erythrocyte membranes during terminal differentiation [52]. *Mthfd2* is known to encode an enzyme involved in mitochondrial folate one-carbon metabolism, which is also recognized as an NAD-dependent mitochondrial methylenetetrahydrofolate dehydrogenase-cyclohydrolase (NMDMC). *Mthfd2* is reported to play a role in embryonic development and pluripotent stem cells in mice [53,54] and is associated with a poor prognosis in hematological and solid cancers [55]. *Isg15* is involved in modulating the development of erythroid differentiation [56]. To our knowledge, the function of *Gm32051* remains unknown. Interestingly, the dramatically increased expression of these genes with unknown functions related to EPO may represent new parameters for the ABP.

A previous study reported the findings obtained after administration of recombinant hEPO (rhEPO) to humans and transcriptome analysis using whole-blood RNA [57]. The RNAs of ABP parameter candidates found in that study [57] were also investigated in detail in other studies and could be recognized as robust biomarker RNA candidates for detecting rhEPO-administered doping [58,59]. In this study, since the amount of circulating EPO hormone increased in our mouse model, we assumed that the RNAs in whole-blood samples from the model were showing the same fluctuations as those reported in the studies described above. The total RNA-seq results were largely consistent with the expression patterns described in previous reports [57,58,59] (Supplementary Figure S6). In particular, five genes (*Bcl2l1*, *Epb42*, *Slc4a1*, *Tmod1*, and *Trim58*) that were analyzed as ABP candidate parameters in the previous study by Wang et al. [58] showed a clear increase of about 2–4 times the average value, which was statistically significant (Supplementary Figure S6). These results suggest that our mouse model partially mimics the physiologic mechanism of rhEPO doping in humans and that the model may be useful for the development of several gene-doping methods. In addition, as mentioned earlier, we found four dramatically upregulated genes (*Apol11b*, *Mthfd2*, *Gm32051*, and *Isg15*) in this model. Since these genes have not been listed in the previous studies [57], they may be genes that specifically increase when AdV-h*EPO* is injected. Because these genes show a drastic increase, they can be considered as better parameters for the ABP; however, these findings should be validated in human studies using AdV-h*EPO*.

We also conducted long-term measurements of the RNAs identified as indirect proof, and the results showed that the expression levels of these RNAs significantly fluctuated for 15 to 30 days after injection of rAdV-h*EPO*. Therefore, these RNAs may be sensitive to the injection of the rAdV-h*EPO* vector and could be used as novel parameters for the ABP. However, it is unclear whether the same results would be obtained in humans. Therefore, future studies should aim to conduct similar experiments on human participants. In addition, since it is also unclear why these RNAs sensitively responded to the rAdV-h*EPO* injection, an additional line of research should aim to elucidate the molecular-level biological mechanisms in tissues and blood after injection of rAdV-h*EPO*.

Currently, automation of inspections is being actively pursued in the medical field. Automation reduces human error, making testing more accurate. On the other hand, in this study, even though the control and Ad*EPO* mice were completely separated during the experimental process, fragments of the hexon region were detected in the blood of the control mice. This form of artificial cross-contamination can influence the results and must be avoided when conducting tests for humans. To avoid the abovementioned cross-contamination, we tried to apply an automation system such as Maholo, which uses an experimental robot similar to LabDroids [60] for gene-doping testing. An automation system can perform its programmed contents with high precision in a clean environment free from artificial contamination. Therefore, an automatic system is expected to be effective in preventing deception on the part of the inspector, yielding a more rigorous inspection system.

This study had some limitations. First, our results identified vector fragments and multiple potential RNA markers as proof of gene doping in the mice model. However, these findings can only be adapted within the mice model. In particular, it is unknown whether the four genes discovered in the RNA-seq under the ABP concept show similar changes in human athletes. The hematopoiesis-related functions of these four genes in humans and mice are also unknown. If these four RNA markers are to be applied to gene-doping tests on human athletes, these tests should be able to avoid the effects of confounding factors that increase hematopoiesis, such as hypoxia, bleeding, and chemical stimulation. Nevertheless, our findings have the potential to expand the research on gene-doping tests. In particular, the animal model developed in this study may be useful to develop several gene-doping tests. This is because the dynamics of nucleic acids can be reflected by the metabolic actions of the living body. In addition, the primer-probe sets and PCR conditions developed in this study would be directly applicable to human testing for gene doping. If research proceeds using this model and humans in the future, quantifying the DNA fragments and RNA expressions that provide proof for gene doping could help identify the date and time on which gene doping was performed by athletes. Thus, despite the multiple limitations associated with the application of the findings of this study to the testing of athletes, the results may be of great significance for the further development of gene-doping tests. Second, evaluation of the hematological parameters influenced by rAdV-h*EPO* until day 30 was not performed. According to our results, because the viral DNA fragments and potential RNA markers serving as proof of gene doping were detected even on day 30 in some mice, it is possible that hematopoiesis was also upregulated over 30 days in the mice. If the characteristics of the long-term hematopoietic effects in the model mice are revealed, the results would facilitate the development of gene-doping tests.

## 5. Conclusions

In summary, our study demonstrated the establishment of a gene-doping mouse model using an rAdV-h*EPO* vector, and we showed that single parameters of the gene-doping model could be evaluated as direct and indirect proof of gene doping by using DNA and RNA analyses of one-drop whole-blood samples over the long term. Moreover, we showed that the overall expression profiles of RNAs significantly changed in whole blood in the gene-doping model and could become new parameters for the ABP. These achievements provide new insights for further technical improvement of gene-doping testing in human athletes.

## Data Availability

The total RNA-seq data have been deposited in the “Gene Expression Omnibus (https://www.ncbi.nlm.nih.gov/geo/)” under accession number: GSE178336.

## Supplementary Materials

The following are available online at https://www.mdpi.com/article/10.3390/genes12081249/s1, Figure S1. Blot raw data for the detection of total EPO in the liver, Figure S2. Blot raw data for GAPDH expression in the liver, Figure S3. No hematopoietic effect of the rAdV unit alone in mice, Figure S4. GSEA predicting the detailed mechanisms of hematopoiesis induced by rAdV-h*EPO* in whole blood, Figure S5. TSEA predicting hematopoiesis in tissue status as reflected by gene expression profile, Figure S6. Total RNA-seq showed reproducibility with the findings of previous studies; Figure S7. Confirmation of the amplicon size and gel cutting before the Sanger sequencing. Table S1. Primer and probe sequences.
