# Supplementary material for: Proof of Gene Doping in a Mouse Model with a Human Erythropoietin Gene Transferred Using an Adenoviral Vector
Source: Genes (Basel). 2021 Aug 16;12(8):1249. doi: 10.3390/genes12081249 (PMC8392868; doi:10.3390/genes12081249)
Supplement: Supplementary file 1 [file genes-12-01249-s001.zip › Supplementary_Figure_S1-7_0805.pdf]

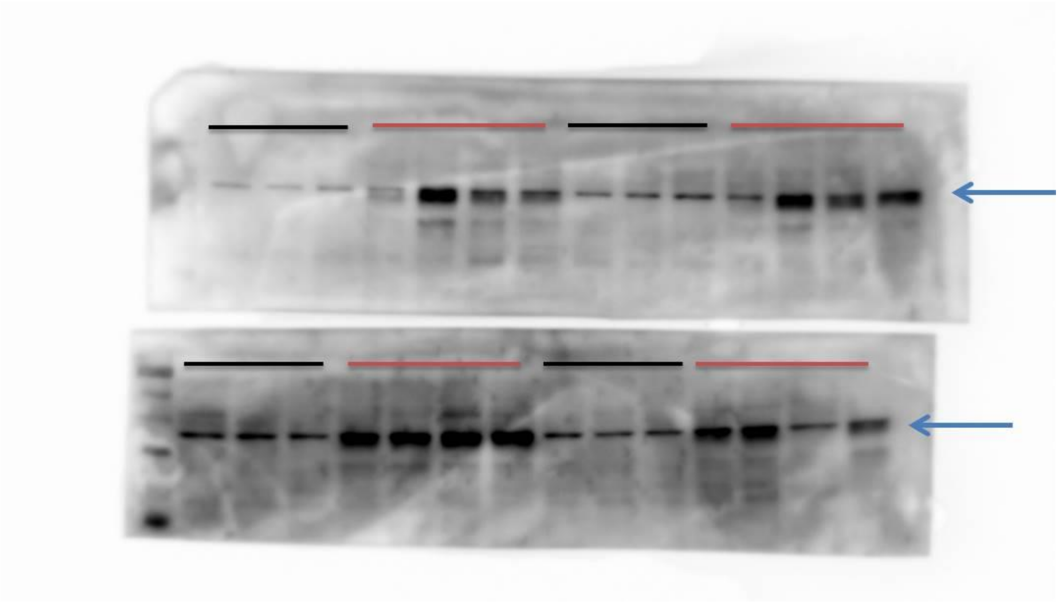

Figure S1. Raw blot data for detection of total EPO in the liver.

The lanes under the black line are for the control mice (n = 12), and those under the red line are for the Ad*EPO* mice (n = 16). The light blue arrow indicates the position of the target bands.

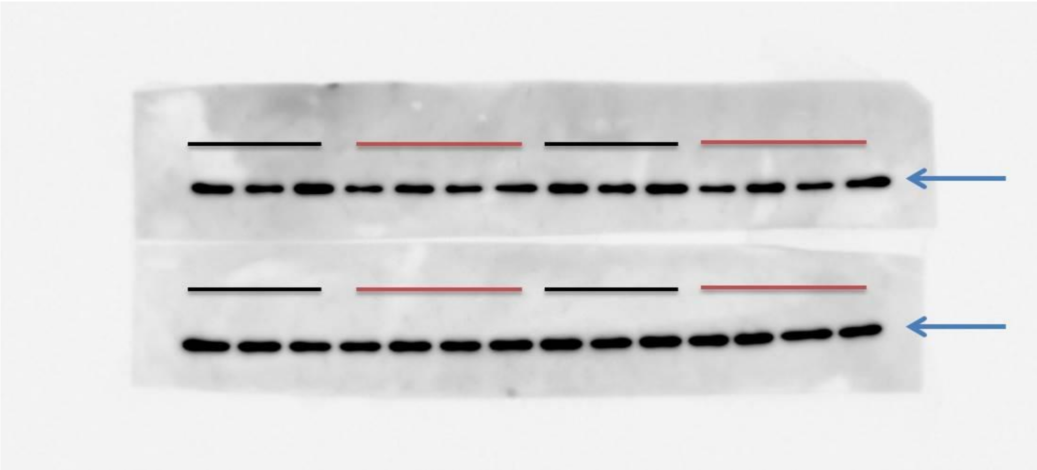

Figure S2. Raw blot data for GAPDH expression in the liver.

The lanes under the black line are for the control mice (n = 12), and those under the red line are for the Ad*EPO* mice (n = 16). The light blue arrow indicates the position of the target bands.

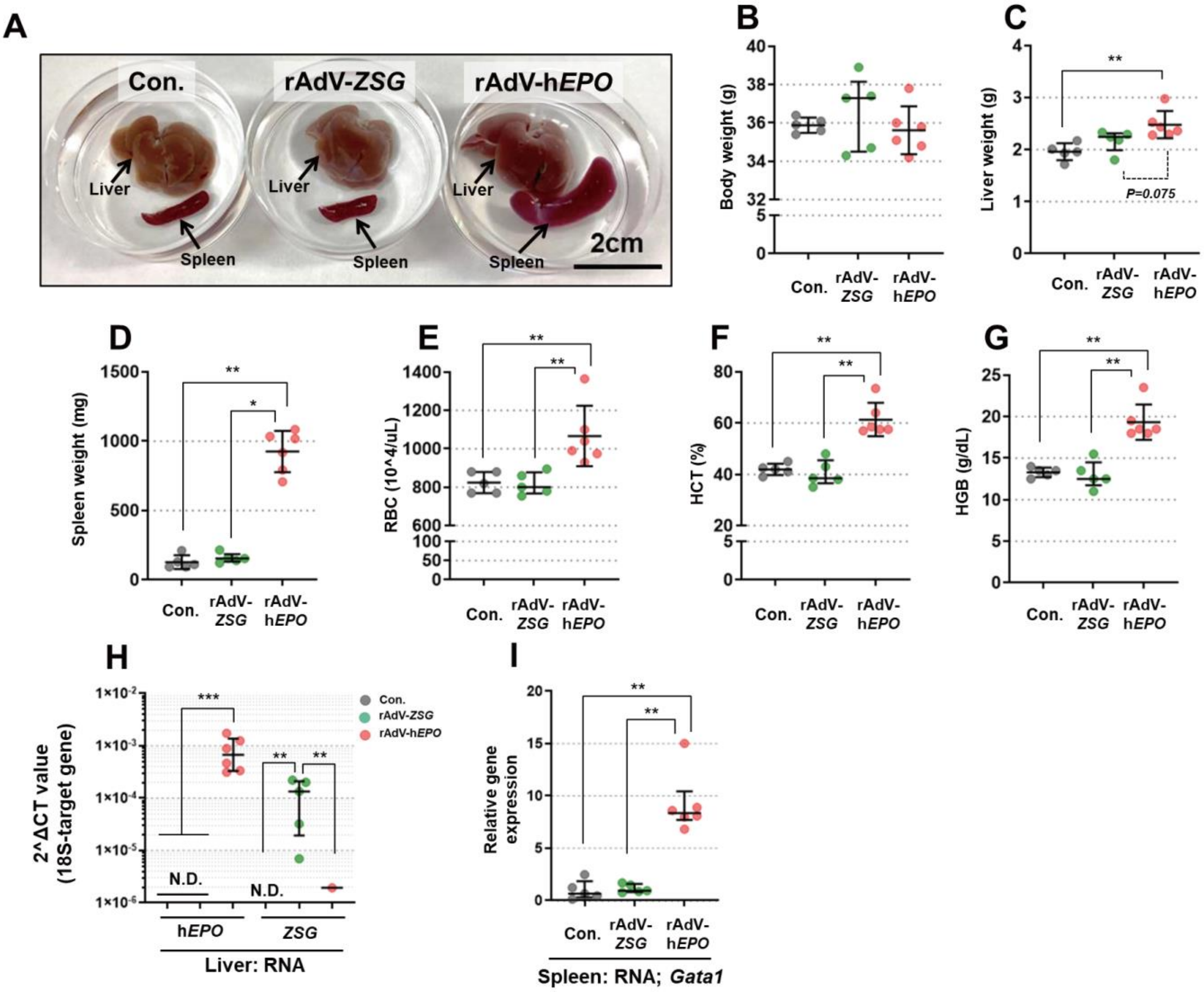

Figure S3. The rAdV unit alone had no hematopoietic effect in mice.

The phenotypes and parameters for hematopoiesis were measured in the Con. (n = 5), rAdV-*ZSG* (n = 5), and rAdV-h*EPO* (n = 6) groups. (A) Representative photographs of the livers and spleens of mice, (B) body weights of the mice, (C) liver weights, (D) spleen weights, (E) RBC counts (10^4/μL), (F) HCT (%), (G) Hgb level (g/dL), (H) gene expression levels of h*EPO* and *ZSG* in the liver shown using the TB Green qPCR assay, and (I) gene expression of *Gata1* in the spleen. N.D.: not detected. *$p < 0.05$, **$p < 0.01$, ***$p < 0.001$.

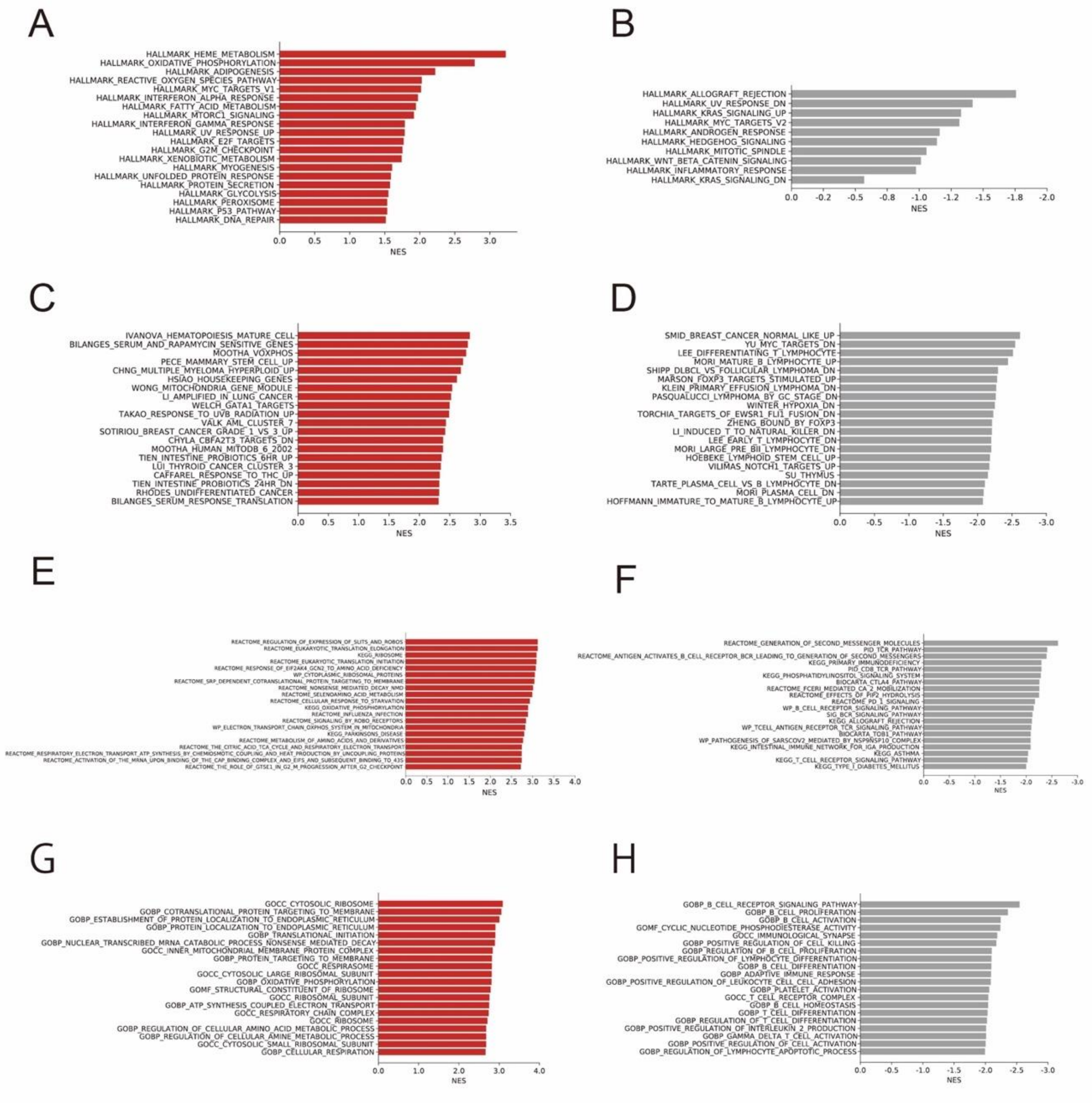

Figure S4. GSEA predicting the detailed mechanisms of hematopoiesis induced by rAdV-h*EPO* in whole blood.

Bar plots showing the top 20 significant MSigDB gene sets in the GSEA results: h.all.v7.4.symbols list of the upregulated (A) and downregulated (B) genes in rAdV-h*EPO*. C2.cgp.v7.4.symbols list of the upregulated (C) and downregulated (D) genes in rAdV-h*EPO*. C2.cp.v7.4.symbols list of upregulated (E) and downregulated

(F) genes in rAdV-h*EPO*. C5.go.v7.4.symbols list of upregulated (G) and downregulated (H) genes in rAdV-h*EPO*. Values are reported as the normalized enrichment score (NES).

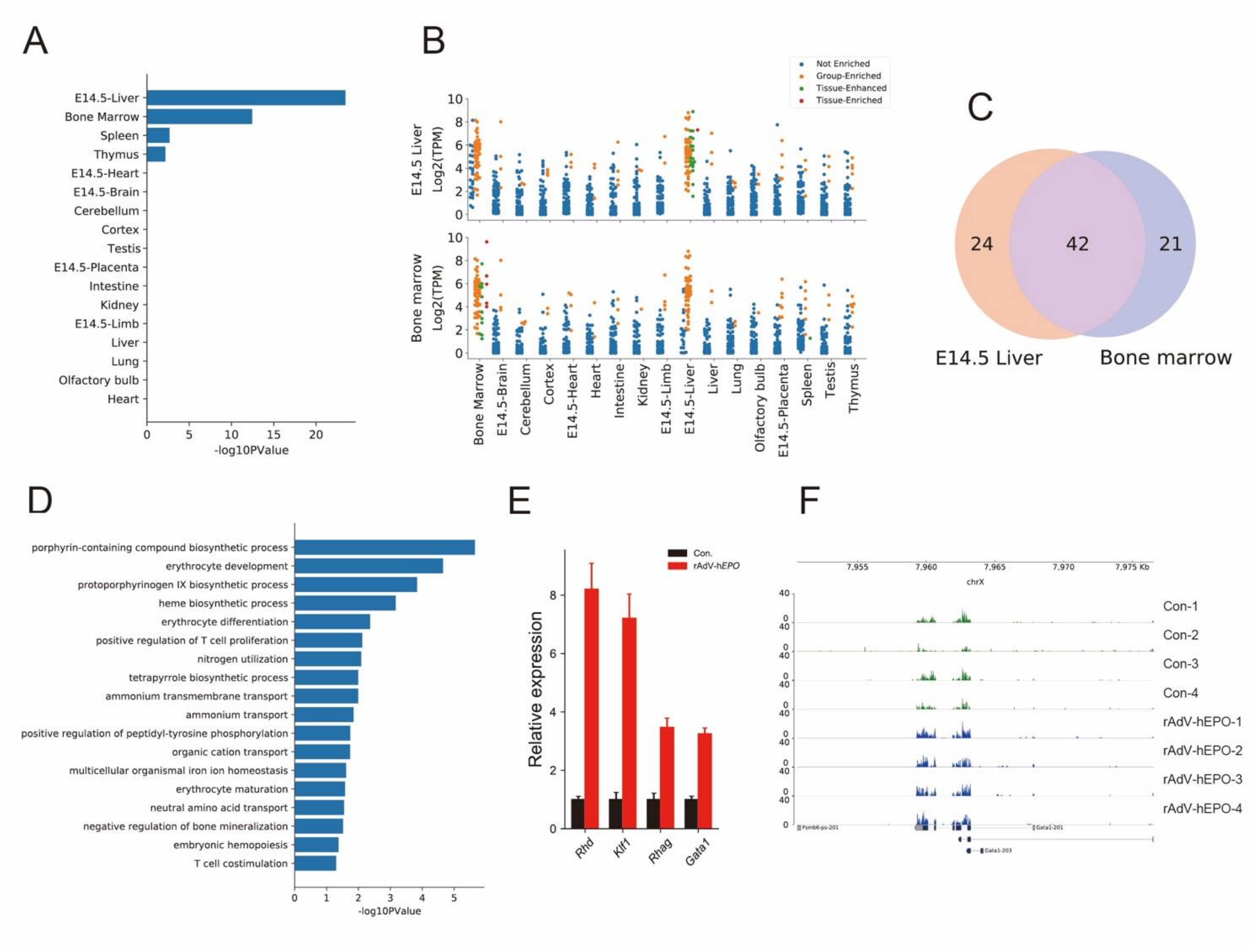

Figure S5. TSEA predicting hematopoiesis in tissue status as reflected by the gene expression profile.

(A) TSEA showing significantly enriched tissues of DEGs. (B) Dot plots showing tissue gene expression of E14.5 liver- and bone marrow-enriched genes in each tissue. (C) Venn diagram indicating DEGs annotated in E14.5 liver and bone marrow. (D) Enrichment analysis by DAVID showing significantly enriched terms in the 42 overlapping genes from Fig. S5C. (E) Relative gene expression (Con. = 1.0) showing erythrocyte development terms in Fig. S5D. Error bars indicate the SEM. (F) Genome browser indicating the read mapping results of the *Gata1* genome region.

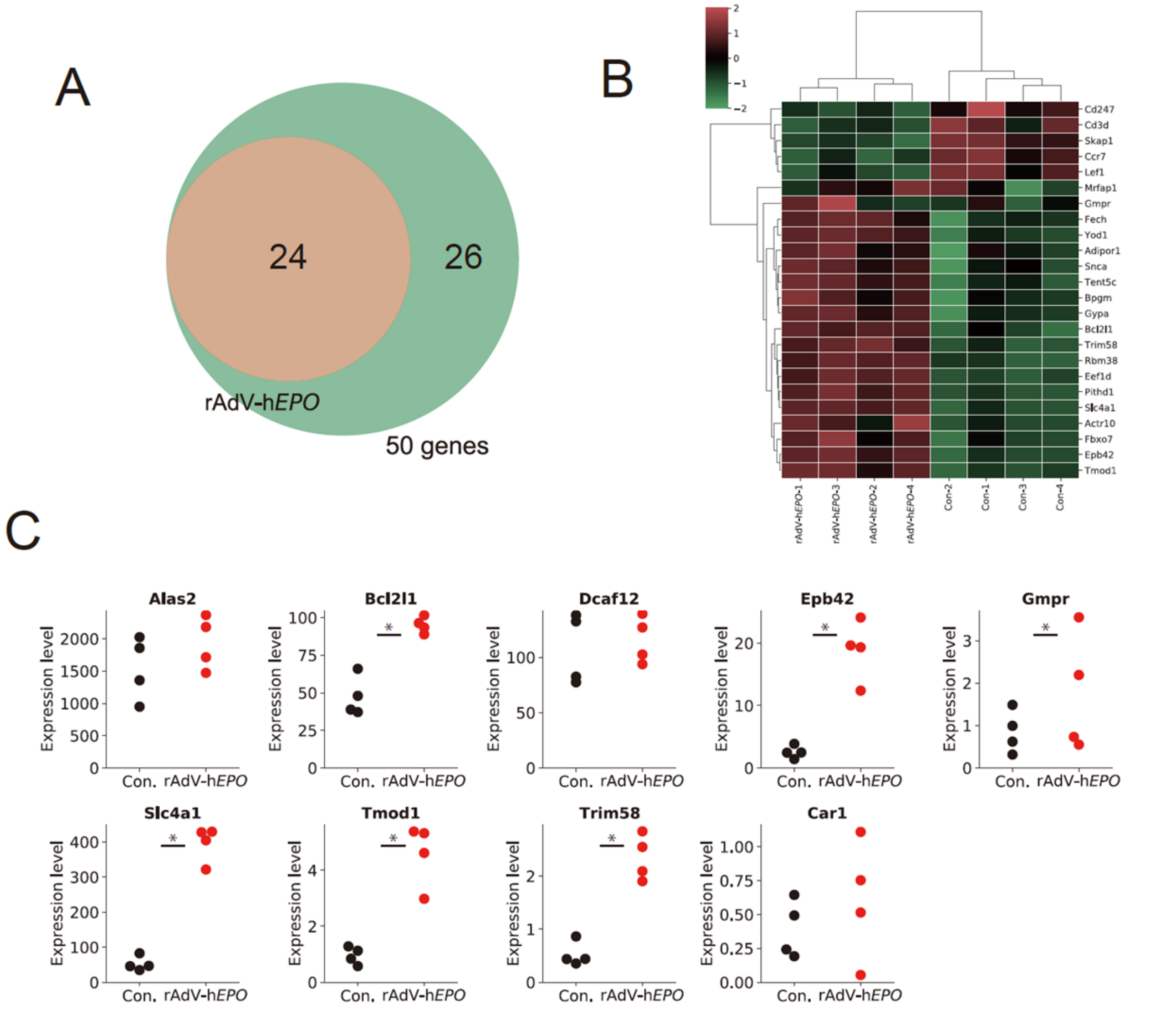

Figure S6. Total RNA-seq showed reproducibility with the findings of previous studies.

Figure S6. Total RNA-seq showed reproducibility with the findings of previous studies. (A) Venn diagram showing overlapping DEGs with a previous study [57]. DEGs were selected from 50 mouse genes converted to human gene names on the basis of a previous study. DEGs were selected by the cutoff with FDR *p*-value < 0.05. (B) Heatmap indicating the expressions of the 24 overlapped genes. (C) Dot plots showing gene expression levels selected from previous studies [57,58,59]. *FDR *p*-value < 0.05.

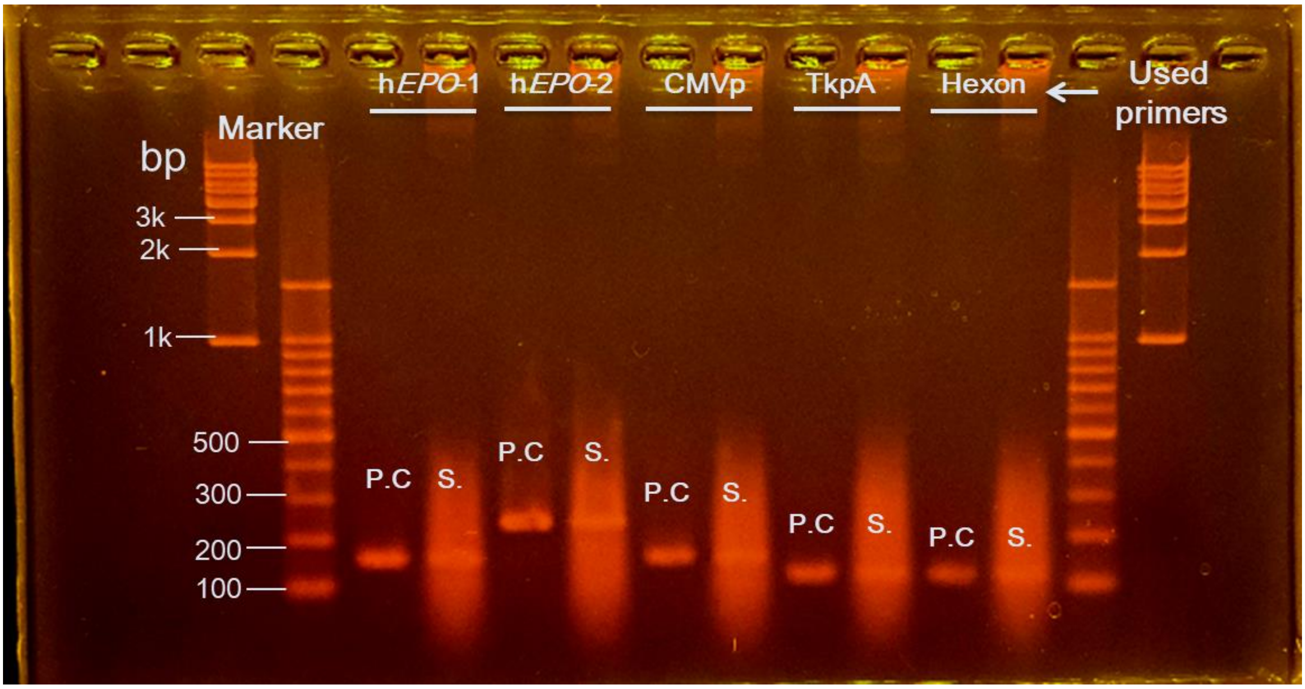

Figure S7. Confirmation of the amplicon size and gel cutting before the Sanger sequencing.

The bands at the same position on the positive control sample were cut out, and subjected to Sanger sequencing. P.C.: Amplicons of the positive control that were amplified form the plasmid to make the standard curve; S.: pooled amplicons that were amplified form the DNA of the Ad*EPO* mice
